# 

**Published:** 1914-08

**Authors:** 


### ANNOUNCEMENTS AND CORRECTIONS

---

Owing to the absence from town of our Associate Editor, Hon. Henry W. Hill, the discussion of laws recently enacted by the State of New York and applying to physicians, will be postponed till the September issue.

